# Globalization of leptospirosis through travel and migration

**DOI:** 10.1186/s12992-014-0061-0

**Published:** 2014-08-12

**Authors:** Medhani Bandara, Mahesha Ananda, Kolitha Wickramage, Elisabeth Berger, Suneth Agampodi

**Affiliations:** 1 Department of Community Medicine, Faculty of Medicine and Allied Sciences, Rajarata University of Sri Lanka, Saliyapura, Sri Lanka; 2 International Organization for Migration, Colombo, Sri Lanka; 3 Medical School for International Health (MSIH), Faculty of Health Sciences, Ben-Gurion University of the Negev, Beersheba, Israel; 4 Tropical Disease Research Unit, Faculty of Medicine and Allied Sciences, Rajarata University of Sri Lanka, Saliyapura, Sri Lanka

**Keywords:** Leptospirosis, Travel, Migration

## Abstract

Leptospirosis remains the most widespread zoonotic disease in the world, commonly found in tropical or temperate climates. While previous studies have offered insight into intra-national and intra-regional transmission, few have analyzed transmission across international borders. Our review aimed at examining the impact of human travel and migration on the re-emergence of Leptospirosis. Results suggest that alongside regional environmental and occupational exposure, international travel now constitute a major independent risk factor for disease acquisition. Contribution of travel associated leptospirosis to total caseload is as high as 41.7% in some countries. In countries where longitudinal data is available, a clear increase of proportion of travel-associated leptospirosis over the time is noted. Reporting patterns is clearly showing a gross underestimation of this disease due to lack of diagnostic facilities. The rise in global travel and eco-tourism has led to dramatic changes in the epidemiology of Leptospirosis. We explore the obstacles to prevention, screening and diagnosis of Leptopirosis in health systems of endemic countries and of the returning migrant or traveler. We highlight the need for developing guidelines and preventive strategies of Leptospirosis related to travel and migration, including enhancing awareness of the disease among health professionals in high-income countries.

## Introduction

Leptospirosis has traditionally been described in the medical literature as a treatable zoonotic disease endemic to low-income countries in temperate and tropical regions [1]. As a clinical entity it is strongly associated with regional occupational and environmental exposures [2]. WHO Leptospirosis Burden Epidemiology Reference Group (LERG) estimates [3], 873,000 annual cases and 48,000 deaths due to leptospirosis [4]. The countries with the highest reported incidence are located in the Asia-Pacific region (Seychelles, India, Sri Lanka and Thailand) with incidence rates over 10 per 1000,000 people s well as in Latin America and the Caribbean (Trinidad and Tobago, Barbados, Jamaica, El Salvador, Uruguay, Cuba, Nicaragua and Costa Rica) [5].

In recent years however, recreational exposure to water has emerged as a strong risk-factor for disease transmission [6]. Over the past twenty years, leptospirosis cases diagnosed among international travelers have become increasingly common [7] and the disease is now emerging as a major public health concern worldwide [5]. Both neglected and under-reported, there is scant epidemiological data on leptospirosis therefore masking the true scope of disease prevalence and making reliable morbidity and mortality statistics difficult to ascertain [3]. Chronic underreporting of leptospirosis is due in large part to clinical misdiagnosis [8]. Patients present with clinical signs difficult to distinguish from other endemic illnesses including dengue fever, malaria, HIV, rickettsial disease and yellow fever. Further, regional and district level health centers often lack appropriate diagnostic laboratories to perform the serological testing required to establish a diagnosis [9].

To date, studies investigating leptospirosis have described high disease prevalence among economically marginalized populations in endemic regions. Urban slums [10]-[15] lacking access to adequate sewage disposal and water treatment infrastructure as well as rural farming communities working in rice [16]-[20] or sugar cane fields [21]-[23] have been highlighted as communities facing the highest risk. These populations are particularly vulnerable to seasonal monsoons and flooding which can heighten the risk of transmission and lead to outbreaks and possible epidemics. Additionally, high-risk occupational groups for the disease include fishermen [24], sewer workers [25], dairy industry workers [26], veterinarians [27], miners [28] and military personnel [29]-[32]. In countries where disease is regionally endemic, internal migration patterns and exposures are crucial risk factors for the spread of the disease.

The rise in global travel and eco-tourism [33] has led to dramatic changes in the epidemiology of leptospirosis. Despite decreasing prevalence of leptospirosis in endemic regions, previously non-endemic countries are now reporting increasing numbers of cases due to recreational exposure [34]. International travelers engaged in adventure sports are directly exposed to numerous infectious agents in the environment and now comprise a growing proportion of cases worldwide. Similarly, in recent years outdoor athletic events have been linked with several outbreaks among foreign travelers.

To date, the global impact of international travel and migration as a major determinant of leptospirosis transmission has yet to be thoroughly analyzed in the medical literature. In this paper we aim to evaluate the impact of human migration and recreational travel on the re-emergence of leptospirosis.

## Methods

### Search strategy

We conducted a comprehensive review of existing leptospirosis literature via online databases. Our principle aim was to identify all studies published in peer-reviewed journals and indexed in PubMed that explored the association between human migration and leptospirosis published through March 2013. Although non-English studies were excluded, we extracted data from studies that had English abstracts. To identify relevant articles not found in PubMed, we supplemented the search strategy by first searching the indices of several journals manually: *Travel Medicine and Infectious Disease*, *Journal of Travel Medicine, International Medical Travel Journal, International Journal of Travel Medicine and Global Health,* and second by reviewing reference lists of primary studies.

### Inclusion criteria and definitions

Studies that reported confirmed and probable cases of leptospirosis were deemed eligible for inclusion. Reviewers screened citations by reviewing titles and abstracts to identify potentially relevant studies. Disagreements between the reviewers were resolved by consensus. The database was then screened a second time using more focused inclusion criteria to isolate only primary articles. Subsequently the full text of each citation was obtained and reviewed. In cases where full articles could not be obtained, the article abstract was used. We adopted the case-definition of leptospirosis based on the guidelines from the LERG [35] to include studies in the review. No restrictions were made with regard to the outcome variables as this was a review of observational studies.

### Data extraction

A data collection form was prepared and pilot tested prior to data extraction activities begun. In order to minimize biases in data extraction, the methods of selected studies were reviewed in detail by two independent reviewers under the supervision of the principle investigator. Subgroup analyses were done to minimize heterogeneity across studies. Data were pooled only when studies were reasonably consistent in their methods. Variables were examined in detail, including: number of cases, country of residence, exposure country/region, proportion of travel related cases, type and nature of study (population based vs. hospital based cohort), type of exposure, time period and year.

### Results

**Figure 1:**
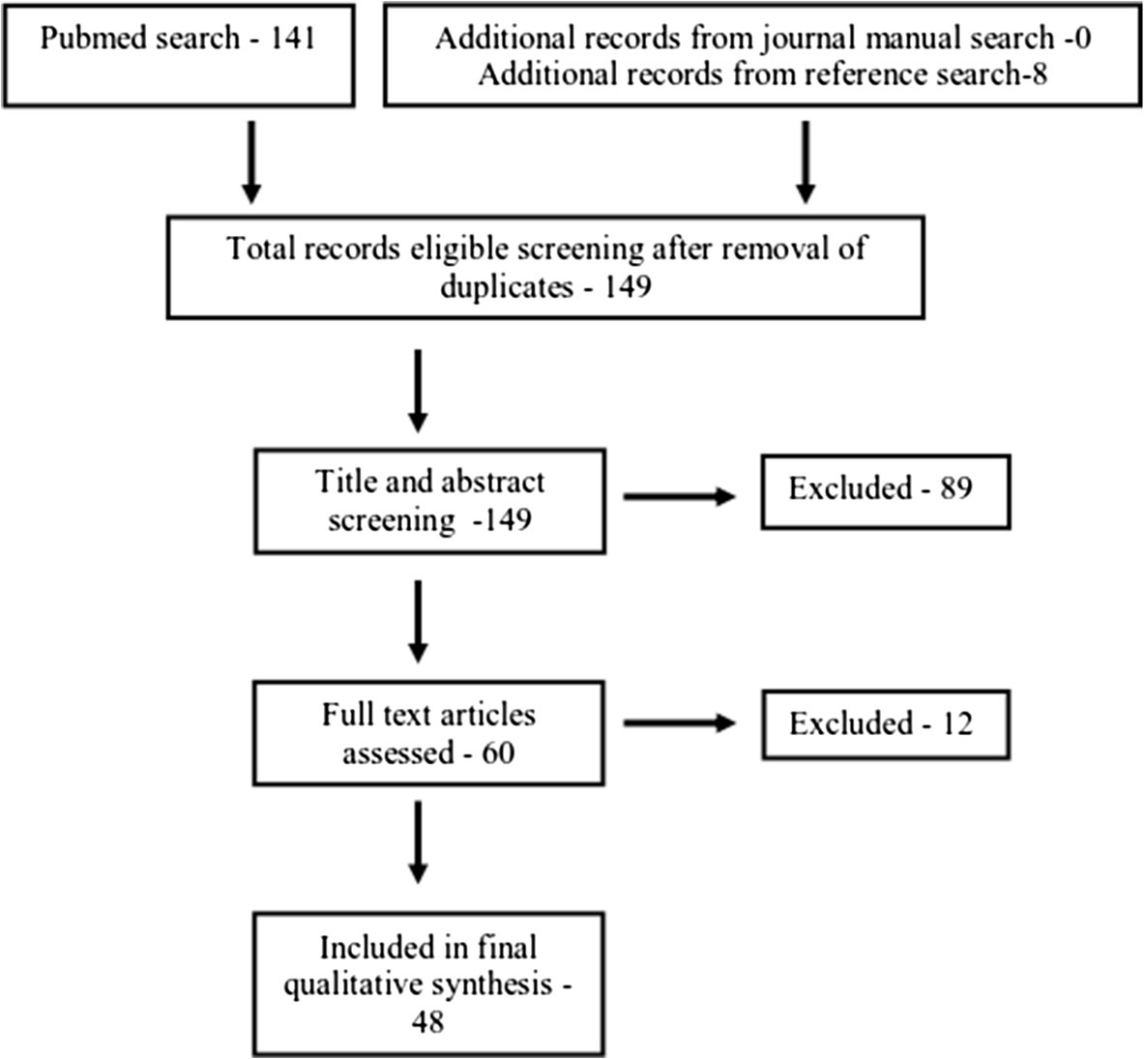
Selection of studies for review.

Of the 10,289 articles we identified on leptospirosis, only 141 met the key-word criteria related to travel/migration. We identified a total of 149 potentially relevant citations from both the PubMed and cumulative literature search once duplicate references were removed. These were further reduced to 60 after undertaking a title and abstract review. Following an in-depth review of the full-texts of these papers 48 were included for final analysis (Figure 1).

Of the 54 publications, 33 reported individual travel reported leptospirosis cases or case reports, nine reported cases by country and/or regional caseload and six reported cases in the context of travel related fever. After the full text review 12 articles were excluded from final qualitative analysis. We excluded a case acquired due to an imported animal, five reports without primary data and six reviews from the final qualitative data analysis. All studies we encountered clearly identified the geographical region where disease was acquired.

Data extraction was completed for 3 distinct subgroups:

1. Papers citing travel as a significant contributory factor to overall country level leptospirosis case loads (9 studies)

2. Those that highlighted the contribution of leptospirosis to larger trends in travel associated fever (5 studies); and

3. Case reports and case series illustrating travel-associated leptospirosis (33 studies)

The nine studies reporting cases of leptospirosis among travellers or migrants as a proportion of the general population contained data of 6 countries: The Netherlands [36],[37], Israel [38], Germany [39],[40], Austria [39], Japan [41],[42], Thailand [43] and the USA [44] (Table 1), all of which are high-income nations with the exception of Thailand. In the USA, country level data was not available, but complete data set for Hawaii was available only up through 2008.

**Table 1:** Country level data and proportion of travel related leptospirosis

**Reference**	**Country**	**Period of study**	**Total cases**	**Travel associated cases**	**Country of exposure**	**Type of exposure**	**Positive test that was diagnostic of leptospirosis**
Goris, Kimberly et al., [36]	Netherlands	1925 - 2008	2553	318(12.5%)	Europe – 132	Recreational- 257,	Culture/MAT
					Asia – 134		
					South America – 25	Job-related 21,	
					Central and North		
					America – 13	Accidental 22,	
					Sub-Saharan Africa – 7		
					Middle East – 2		
					Australia – 1		
Leshem et al., [38]	Israel	2002-2008	48	20(41.7%)	Southeast Asia - 15,	Participation in water related activities	Not mentioned
					Africa - 2,		
					Oceania - 2, Central		
					America - 1		
Hoffmeister et al., [39]	Germany and Austria	1998-2008	59	24(40.7%)	Caribbean - 10,	Recreational	ELISA, MAT, PCR
					Asia - 9, Eastern Europe - 3, Central America - 1,		
					South America - 1		
Taniguchi et al., [42]	Japan	1999-2008	95	7(7.4%)	Not mentioned	Not mentioned	Not mentioned
Jansen et al., [40]	Germany	1997-2003	248	39(15.7%)	France - 4,	Not mentioned	Not mentioned
					Greece - 2,		
					Poland - 2,		
					Hungary - 2,		
					Norway-1,		
					Croatia-1,		
					Bulgaria-1		
Narita et al., [41]	Japan	1999	14	9(62.3%)		Exposure to contaminated soil or water	Not mentioned
Ariyapruchya et al., [43]	Thailand	1994 - 2000	59	10(16.9%)	Northeastern part of Thailand		
Katz et al., [44]	USA	1974 - 1998	752	43(5.7%)	43 - outside the state		
					27 - Federated States of Micronesia,		
					6 -American Samoa,		
					4 - Guam,		
					from Costa Rica-2		
					Thailand-1,		
					Philippines-1		
					Mexico-1		
					Utah-1		
Crevel et al., [37]	Netherlands	1987 - 1991	237	32(13.5%)	Thailand -24	Contact with freshwater,	MAT,ELISA
					Other Southeast Asian countries-4,	21- water-rafting in Thailand	
					Ivory Coast-1,		
					Surinam-3		

Among reports of systematically collected country level data, Israel reported the highest incidence of travel associated leptospirosis (41.7%). In Germany, before 2003 this contribution was only 15.7%. However, a later study showed that the travel related cases increased up to 40.7% in 2008. Netherland has the largest reported database describing travel associated leptospirosis which included 318 cases out of 2553 cases related to international travel. In Germany and Netherlands, intra-country travel comprised over 40% of the disease burden. One study conducted in Germany using aggregate data from 1962–2002 found that 13 out of 39 travel related leptospirosis cases were acquired from European countries where leptospirosis is rare, with the majority of these cases occurring between 1960 and 1980. The study results suggest that in the last two decades, this trend has shifted, with Latin America and Caribbean Islands serving as the main geographical location for disease transmission and infection among German nationals. Data from Japan reports that around 7% are none residents thus travel related. Data from Hawaii shows that 5.7% of leptospirosis cases reported in Hawaii were from mainland USA and 23% from smaller islands. Other than Hawaii islands where Leptospirosis is endemic and disease transmission was within the state, the major contribution to travel related leptospirosis was from southeast Asia/Asia.

Contribution of leptospirosis to travel associated fever was reported among more than 7000 febrile cases in six studies (Table 2). All studies were from developed countries. Leptospirosis was responsible for 0.21% to 2.65% of all travel associated fever cases. Among Western travelers, which included travelers from North America, Europe, Israel, Japan, Australia and New Zealand, leptospirosis contributed to 2.65% of all travel related febrile illnesses [45]. Individual reports from Sweden [46], Australia [47] and Finland [48] and combined reports by Flores-Figueroa et al. [49] and Field et al. [50] showed less than 1% contribution. South East Asia was reported as the main exposure area.

**Table 2:** Leptospirosis cases detected out of travelers presenting with fever

**Reference**	**Country**	**Period of study**	**Total cases**	**Total fever cases**	**Country of exposure**	**Type of travel**
Jensenius et al., [45]	North America, Europe, Israel, Japan, Australia, New Zealand	1996 – 2011	88(2.65%)	3326	Caribbean - 7, Central America - 15, North Africa - 3, Oceania - 2, South America - 4, South Central Asia - 3, Southeast Asia - 49 (Thailand - 19, Laos - 11), Sub-Saharan Africa - 5	Tourists-82% Others; on business and visiting friends and relatives
Flores-Figueroa et al., [49]	Canada - 1	1996-2010	6(0.75%)	804	Costa Rica, Mexico, Panama	Tourist, missionary and business
	USA – 4					
	France - 1					
Siikamaki et al., [48]	Finland (Helsinki)	2005 – 2009	1(0.21%)		Asia	Unknown
Field et al., [50]	Europe	2008	7(0.50%)	1378	Cambodia-2, Cameroon-1, Central African Republic-1, Costa Rica-1, Indonesia-1, Reunion-1	Unknown
Askling et al., [46]	Sweden	2005-2008	7(0.50%)	1432	Tropical countries - Africa, Asia, America	Unknown
Goldsmid et al., [47]	Australia (Tasmania)		1	NR	India	Studies (river)

Of the 27 case reports and 6 case series, 3 reported on leptospirosis due local travel within the country. Two of these studies examined transmission from the continental US to Hawaii [51],[52] and from the Jordan River Valley in Israel to the northern part of the country [32]. All other case reports were focused on infection secondary to travel outside of the patients’ country of residence (Table 3) which included 11 countries; Germany (7) [53]-[59], Netherlands (5) [60]-[64], France (5) [65]-[69] Australia (3) [70]-[72], USA (4) [73]-[77], Israel (1), Canada (1) [78], Norway (1) [79], Italy (1) [80], Japan (1) [81], and Sweden (1) [82]. Leading exposure countries were from South East Asian region, including Thailand, Malaysia and Philippines which accounted for more than 1/3rd of cases. All case reports and case series were from high income countries.

**Table 3:** Case reports and case series on travel related leptospirosis

**Reference**	**Country**	**Country of exposure**	**Region/City**	**Disease confirmation**
Walter et al., [54],	Germany	Canada		
Grobusch et al., [53]	Germany	Dominican Republic	Playa Dorada, Santiago/Los Ciruelitos, Santa Domingo, Juan Dolio, Mao	ELISA
Teichmann et al., [59]	Germany	Philippines		MAT
Green and Busuttil, [58]	Germany	Sardinia		
Bernasconi et al., [57]	Germany	Switzerland	Southern	
Steffens et al., [56]	Germany	Thailand		Serology, PCR
Seilmaier and Guggemos [55]	Germany	Thailand and Laos		Serology
Gelman et al., [73]	USA	Ecuador, Costa Rica		Culture, Dark field microscopy
Duplessis et al., [51]	USA	Hawaii	Maunuwili falls	
Pashkow et al., [75]	USA	Honduras		
Haake et al., [76]	USA	Malaysia	Sabah	Dip-S-Ticks, PanBio ELISA, Culture
Mortimer [74]	USA	Malaysia	Sarawak	
Coursin et al., [52]	USA	USA	Hawaii	MAT
Monsuez et al., [67]	France	Africa	Cote d’Ivoire (Ivory Coast)	ELISA
Perret et al., [68]	France	Gabon		IgM
Jaureguiberry et al., [66]	France	Ivory Coast - 1, China - 1		ELISA, MAT
Simon et al., [69]	France	Mauritius Island		ELISA, MAT, PCR
Arzouni et al., [65]	France	Portugal and Spain		Dark field microscopy, PCR, Western blot, Culture
Maldonado et al., [64]	Netherlands	China		MAT
Arcilla et al., [60]	Netherlands	Dominican Republic	Altos de Chavón	MAT, ELISA, PCR
Wagenaar et al., [63]	Netherlands	Malaysia	Langkawi island	ELISA, MAT
Helmerhorst et al., [61]	Netherlands	Thailand	Bangkok and the North of Thailand	MAT
Kager et al., [62]	Netherlands	Thailand		Serology Culture
Heron et al., [70]	Australia	Africa, Ghana	Ghana	
Gill et al., [71]	Australia	Fiji		ELISA (IgM)
Korman et al., [72]	Australia	Indonesia	Kalimantan	MAT, Culture, Dark field microscopy
Hadad et al., [32]	Israel	Israel	Jordan river (Nothern Israel)	MAT
Paz et al., [77]	Israel	Thailand, Cambodia	Kochang island, Thailand	MAT
Leung et al., [78]	Canada	Malaysia	Northeastern Malaysia	ELISA, MAT
Lagi et al., [80]	Australia	Italy	Venice	PCR, MAT
Sakamoto et al., [81]	Japan	Indonesia	Bali island	Dark field microscopy, MAT
Myrstad et al., [79]	Norway	France	Southern France	IgM, IgG
Guron et al., [82]	Sweden	Thailand		Serology

## Discussion

The results from this review suggest a dynamic shift in the epidemiology of leptospirosis transmission due to increased human travel and migration on a global scale. While previous studies have offered insight into intra-national and intra-regional transmission, few have analyzed transmission across international borders. The results presented here suggest that secondary transmission of leptospirosis via human travel and migration across national borders is re-shaping the landscape of disease incidence and prevalence worldwide. Alongside regional environmental and occupational exposure, international travel now constitutes a major independent risk factor for disease acquisition.

Despite increases in travel-associated disease, the overall contribution of diagnosed leptospirosis to cases of febrile illness in returning travelers is still quite low (2.4%) [45]. This discrepancy is likely due to the lack of clinical suspicion among home-country clinicians as leptospirosis is rarely included in the differential diagnosis outside of endemic regions. Additional obstacles to diagnosis include the lack of home-country diagnostic facilities and general diagnostic inaccuracy due to serovar diversity between geographic regions. Countries having the highest number of cases detected in travelers returning from endemic regions (United States, Netherlands, Japan, France, Germany and Australia) are also equipped with highly developed reference laboratories, diagnostic capacity and research facilities. Such capacities may be limited in developing countries where tourist travel to endemic countries is equally common, ultimately leading to poor case detection and reporting bias.

In an era of globalization with increased global travel and migration, diseases that were thought to be isolated to tropical regions or affecting marginalized communities can no longer be considered as ‘tightly contained’ static entities [5]. It is imperative to view new international trends in transmission as a direct outgrowth of globalization. Such diseases are not emerging threats, but rather have been threats all along. Confined to mostly impoverished fishing and rice farming communities, Leptospirosis has for many decades gone largely undetected and neglected. The disease has also been a leading cause of morbidity and mortality in such communities for generations. Although Leptospirosis remains the most widespread zoonotic disease in the world, the greatest burden of the disease still remains with such marginalized communities. They face daily occupational exposure risks in order to earn their livelihoods. The new trends in global transmission stands as an important reminder that it is essential to design and implement a rigorous disease detection, prevention and treatment plan starting first at the community level.

Due to the complexity of the disease transmission, primary prevention of leptospirosis is difficult. In low and middle income tropical countries, the ecological system provides a conducive environment for leptospirosis transmission. Identification of local ‘hot spots’ of leptospirosis may help facilitate preventive activities. Public health authorities in endemic areas should coordinate with regional level authorities to compile and monitor epidemiological trends with the goal of actively identifying geographical areas which would benefit from increased service provision and education. If these hot spots correspond with mass gatherings or tourist attractions, then application of targeted primary and secondary preventive measures may become more feasible. As an example, recent reports of internal travel associated leptospirosis occured in Sri Lanka, in a place where white color workers were engaged in water sport [83]. Interventions were needed to target those involved in organizing recreational travel packages for white-water rafting in such settings. Strategies may also include participant education and chemoprophylaxis to prevent leptospirosis in those registering for such activities. Recent increase in leptospirosis among people engaged in water sports also pose the question of whether chemoprophylaxis should be taken as a routine practice. Since the evidence to support the use of chemoprophylaxis is still poor, more studies are needed to inform decision making [84].

The results of this review highlight the urgent need for developing travel guidelines and preventive strategies of leptospirosis related to travel. Awareness of leptospirosis among health professionals practicing in high-income countries where the disease seems exotic is essential for proper diagnosis and treatment. Further, stake holders such as tour companies, adventure sport organizers should also receive adequate knowledge on the increasing risk of leptospirosis.

Further research is needed to investigate the impact of occupational migration and forced displacement on leptospirosis prevalence and disease distribution. Given that migrant workers and refugees are often the most marginalized and underserved communities, detection, treatment and prevention of leptospirosis among this high-risk cohort has been largely ignored to date. Lastly additional studies are needed to assess the efficacy of long-term chemoprophylaxis with doxycycline and to identify other alternative less toxic agents for long-term use in high-risk groups.

We undertook our literature review using PubMed as the main electronic database with hand-searches of selected bibliographies to identify additional relevant literatures. This may not have captured an exhaustive list of literature. We did not analyze the disease transmission risk associated with the total length of time traveling, exposure history, route of transmission, visits to multiple regions in succession, occupation and clinical presentation stand as possible confounding variables which should be done in future studies.

## Competing interests

All authors declare (1) no support from any organization for the submitted work; (2) no financial relationships with any organizations that might have an interest in the submitted work in the previous three years; and (3) no other relationships or activities that could appear to have influenced the submitted work.

## Authors’ contribution

MB and MA carried out the literature search, data extraction and table preparation. MB and EB analyzed and interpreted data and prepared the draft manuscript. EB completed the manuscript writing. KW and SA conceived the study, design the methodology, revised the final manuscript and coordinated the review process. All authors read and approved the final manuscript.
